# The impact of physical activity on adolescent mental health status: the mediating effect of school adaptation

**DOI:** 10.3389/fpsyg.2025.1573129

**Published:** 2025-05-15

**Authors:** Menghan Zhang, Chenguang Gu, Hui Zeng, Xiangren Yi

**Affiliations:** ^1^School of Education, Nanning University, Nanning, China; ^2^School of Physical Education, Guangxi University, Nanning, China; ^3^School of Physical Education, Shandong University, Jinan, China

**Keywords:** adolescents, physical activity, school adaptation, mental health status, structural equation modeling

## Abstract

**Objective:**

This study was designed to examine the mental health status of Chinese adolescents, investigate the effects of adolescent physical activity on mental health status, and verify the mediating effect of school adaptation.

**Methods:**

A survey and analysis were conducted on 9,701 high school students in Shandong Province using the Physical Activity Questionnaire for Adolescents (PAQ-A), the School Social Behavior Scale-2 (SSBS-2), and the Symptom Checklist-90 (SCL-90) self-rating scale. The overall status of adolescent mental health was analyzed, regression modeling along with structural equation modeling was used to test whether school adaptation played a mediating role.

**Results:**

The structural equation model was validated as a good fit (GFI = 0.950, RMSEA = 0.048, CFI = 0.972, NFI = 0.970, SRMR = 0.077). Physical activity was significantly and positively correlated with mental health status (*β* = −0.691, *p* < 0.001), school adaptation (*β* = 1.153, *p* < 0.001), and school adaptation was significantly and positively correlated with mental health status (*β* = −0.287, *p* < 0.001). The direct effect of physical activity on mental health status was 0.011 and the indirect effect of physical activity on school adaptation and school adaptation on mental health status was 0.024.

**Conclusion:**

Physical activity was positively related to mental health status, with physical activity significantly and positively predicting mental health status; school adaptation was significantly and positively related to physical activity and mental health status, with school adaptation mediating between physical activity and mental health status.

## Background

Mental health refers to a state of psychological well-being that enables individuals to cope with life’s stresses, realize their abilities, learn, work and contribute to society (World Health Organization, 2021). The impairments in mental health are collectively referred to as mental health problem (Xiu et al., 2022). A global report by the World Health Organization states that one in seven children aged 10–19 suffers from a mental health problem, accounting for 13% of the global burden of disease in this age group (World Health Organization, 2021). Mental health problem has become a significant disease burden among adolescents in China (World Health Organization, 2021). Recent nationwide mental health research indicates that nearly 25% of teens report experiencing mild or severe depression (World Health Organization, 2021). Adolescents with mental disorders are particularly vulnerable to mood disorders (World Health Organization, 2021), conduct disorders (Institute of Health Metrics and Evaluation, n.d.), eating disorders (O’Neil et al., 2014), psychosis (Ezquerra et al., 2024), suicide, self-harm (Younes et al., 2016), and risk-taking behaviors (Lahoti et al., 2024). Therefore, it is crucial to address adolescent mental health issues.

Physical activity (PA) is defined as any movement that results in the exertion of capacity due to the contraction of skeletal muscles (World Health Organization, 2022). The Unified Theory of Physical Activity suggests that engaging in physical activity inherently leads to greater benefits for individuals (Tayebi et al., 2025). Regular participation in physical activities often promotes positive changes in various health-related behaviors (Zheng et al., 2024). Studies have found a significant correlation between youth physical activity and mental health (Zhao et al., 2024). Physical activity enhances blood flow and oxygen delivery to the brain, which improves concentration (Nay et al., 2021). It also boosts adolescents’ self-concept, self-esteem and self-confidence while reducing stress, anxiety and depression (Haverkamp et al., 2020). Appropriate physical activity is important for both prevention (Harvey et al., 2018) and treatment (Stubbs et al., 2017) of mental health, as it can reduce the incidence of issue such as anxiety and depression.

Adolescence is a critical stage for mental health and well-being, a time when young people develop self-control, social interactions and learning skills (World Health Organization, 2023). Mental health status (MH) is the product of many interacting factors. Schools, as social environments, can directly impact individuals as a non-negligible factor influencing adolescents’ adjustment and psychological development (Song et al., 2024). For Chinese high school students, schools are often the first places, apart from family, where adolescent mental health is recognized. Therefore, schools are recognized as one of the key environments for psychological interventions (Xiu et al., 2022).

School adaptation (SA) refers to students’ overall performance in coping with learning tasks, managing peer and teacher relationships, participating in group activities, and regulating emotions (Huang et al., 2023). Emerging evidence highlights SA as a critical mediator between PA and MH. A longitudinal study demonstrated that adolescents with higher PA levels exhibited better SA (e.g., improved peer relationships and emotional regulation), which in turn predicted lower depressive symptoms (Bell et al., 2019). Social-cognitive theory further supports that PA fosters self-efficacy and prosocial behaviors, foundational to successful SA and subsequent MH outcomes (Romeo et al., 2021). Adolescent school adaptation is divided into two areas: social competence and antisocial behavior (Merrell et al., 1993). Studies have found that good social competence, such as positive relationships with parents, teachers, and peers, contribute to a sense of well-being experience (Tomé et al., 2014). Positive peer relationships can reduce negative emotions such as loneliness and improve academic adjustment in adolescents (Kiuru et al., 2024), while good teacher-student relationships can reduce internalizing problems such as depression and anxiety (Wang et al., 2024). In contrast, difficulties in adapting to academic pressures and interpersonal relationships can lead to maladaptation. Statistics indicate that about 18 to 35% of Chinese adolescents experience maladaptation at school (Wang, 2023). Such challenges not only seriously hinder academic progress, also lead to psychological problems (Mei et al., 2019) such as anxiety, impulsivity and depression (Wang et al., 2024). School adaptation results from various factors, including personal characteristics and relationships with classmates and teachers. Thus, the impact of good school adaptation on adolescents’ mental health status is crucial.

Numerous studies have shown that participation in physical activity aids students in adapting to the school environment, increases their capacity for cooperation, and promotes their physical and psychological well-being (Yan et al., 2020). Students who regularly engage in sports activities tend to exhibit favorable psychological factors, such as higher self-esteem and a greater sense of achievement. Furthermore, the positive psychological factors derived from this engagement can enhance adolescents’ school adaptation abilities (Tankersley et al., 2021). In particular, in team sports activities or physical education courses, social interactions and cooperation with teammates can improve students’ interpersonal skills (Eather et al., 2023), foster a sense of belonging, and enhance overall well-being (Opstoel et al., 2020). Therefore, physical activity has a positive impact on an individual’s school adaptation, and physical activity can improve students’ school adaptation.

In summary, preliminary empirical studies suggest that physical activity promotes psychological well-being and improves school adaptation, with school adaptation closely linked to mental health. However, there is limited research on the mediating role of school adaptation between physical activity and adolescent mental health status. This study aims to explore the correlation between physical activity and mental health status in adolescents and verify the mediating effect of school adaptation between physical activity and mental health. The hypothesizes are: (1) physical activity is positively correlated with mental health status; (2) school adaptation mediates the relationship between physical activity and mental health status. A structural equation model will be constructed to synthesize and analyze the role of school adaptation in the relationship between physical activity and mental health status, aiding in efforts to reduce mental health problem among adolescents.

## Methods

### Participants and procedure

Data for this study were obtained from the Database on Youth Health (DYH) of the National Population Health Data Center, a longitudinal database on youth health in China, which is a publicly shared dataset on the health and health-related behaviors of Chinese adolescents and includes comprehensive data from both middle and high school (National Population Health Data Population Health Data Archive PHDA, Shandong University, 2021; Zhang et al., 2022). This study utilized data from 2020 to 2021, selecting 30 high schools from nine cities in Shandong Province using the population proportional sampling (PPS) method. Three high schools were randomly selected from each municipality, with two classes per grade level. Inclusion criteria for the study: children and adolescents aged 12–18 years, without serious physical or mental illness, who voluntarily participated and signed an informed consent form, who possessed basic language and cognitive skills, and who were able to complete the study-related questionnaires or tests independently or under guidance. Exclusion criteria: (1) no physical fitness measurements and questionnaires; (2) severe chronic diseases, motor dysfunction, or other health problems that may affect the study results. A total of 9,701 students completed questionnaires for the study, including 4,536 boys, accounting for 46.8%, 5,165 girls, accounting for 53.2%, more non-only children, accounting for 70.9%, and 6,621 boarding students, accounting for 68.3%.

### Measures

#### Physical activity

Physical activity was assessed using the Physical Activity Questionnaire for Adolescents (PAQ-A), which is a revised version of the Physical Activity Questionnaire for Older Children (PAQ-C) (Kowalski et al., 2004). The questionnaire inquired about activities during the past 7 days, the score of physical activity level was 5 points (1–5 points), and the higher the score was, the higher the level of physical activity was. It can be divided into low physical activity level (1–1.9 points) and high physical activity level (2–5 points) (Adeniyi et al., 2011). The validity and reliability of the PAQ-A has been confirmed in Chinese adolescents (Xiu et al., 2022). With Cronbach’s alpha = 0.835, KMO = 0.882, *p* < 0.001, and *χ^2^* = 25026.452, proving that the scale has good reliability and validity. Those questions asked: which of the following best describes your performance in the past week? “I spend almost all my free time doing activities that have nothing to do with physical activity”; “I sometimes (once or twice in the last week) do some physical activity in my free time (e.g., exercise, running, swimming, cycling, aerobics, etc.)”; “I often (3–4 times in the last week) do some physical activity in my free time”; “I often (5–6 times in the last week) do some sports in my free time”: “I do some physical activity in my free time very often (7 times or more in the last week).”

#### School adaptation

School adaptation was measured using the School Social Behavior Scale-2 (SSBS-2), which assesses social competence and antisocial behavior (Merrell et al., 1993). The Social competence sub-scale includes three dimensions of peer relations (PR), self-management (SM), and learning behaviors (AB), and the antisocial behavior sub-scale includes three dimensions of hostile-irritability (HI), antisocial-aggression (AA), and defiant-disruptive (DD) (Alfonso et al., 2007). Since the questions on the antisocial behavior scale questions were phrased in reverse, they were assigned reverse scores. High scores should represent negative evaluations and low scores represent positive evaluations, which is the opposite of a positive scoring question. Higher scores on the scale’s composite score indicate that students are more school-adaptable. In this study, Cronbach’s alpha = 0.912, KMO = 0.969, *p* < 0.001, and *χ^2^* = 357967.343, demonstrating that the SSBS-2 has good reliability and validity.

#### Mental health status

Mental health status data were assessed by the Symptom Checklist 90-Revised (SCL-90), a self-reported mental health questionnaire designed to screen for a wide range of psychological problems (Liu, 2004). This scale consisting of 90 items, including the following 9 psychiatric symptom factors: somatization, obsessive-compulsive symptoms, interpersonal sensitivity, depression, anxiety, hostility, phobia anxiety, paranoid ideation, and psychoticism. The Global Severity Index (GSI) is calculated as the average of the 9 subscale scores. Higher mean values for each dimension reflect the severity of their poor self-perception. Exploratory factor and correlation analyses across multiple studies indicate that the SCL-90 scale has high reliability and validity (Mei et al., 2019). In this study, the SCL-90 demonstrated good reliability and validity: Cronbach’s alpha = 0.986, KMO = 0.993, *p* < 0.001, *χ^2^* = 605059.802.

### Statistical analyses

SPSS 26.0 software was used for data processing and analysis. Results were expressed as relevant statistical indicators. Significant difference was set at *p* < 0.05. An independent sample *T*-test and a univariate analysis of variance test were used to compare the intergroup differences between physical activity and adolescent mental health status. Multiple linear regression and structural equation modeling were used to analyze the direct and indirect relationships among the variables. In order to verify the applicability of the model, Bootstrap technique in interval estimation was employed to test the mediation effect, confirming mediation when the 95% confidence interval did not include zero (with the sample size of 5,000).

## Results

A total of 9,701 students (15.73 ± 1.53 years) completed all study questionnaires. The demographic characteristics of the participants were summarized in Table 1. Overall, 4,536 male students (46.8%), 5,165 female students (53.2%), with 70.9% being non-only children and 68.3% boarding students.

**Table 1:** Participants’ demographic characteristics.

Demographic characteristics		Number of participants	*n* (%)
Gender	Male	4,536	46.80%
	Female	5,165	53.20%
An only child	Yes	2,823	29.10%
	No	6,878	70.90%
Boarding	Yes	6,621	68.30%
	No	3,080	31.70%

### Effects of different levels of PA on adolescents’ MH

Multivariate analysis of variance (MANOVA) with physical activity as the independent variable and school adjustment as the dependent variable (Table 2). The results indicated statistically significant differences in various physical activity dimensions, such as somatization, obsessive-compulsive symptoms, interpersonal sensitivity, depression, anxiety, hostility, phobia anxiety, paranoid ideation, across different levels of physical activity. *Post hoc* comparisons revealed that adolescents with low levels of physical activity scored higher on all nine dimensions than those with high levels of physical activity (*p* < 0.05).

**Table 2:** Mental health (MH) scores of adolescents at different levels of PA (*x* ± *s*).

Dimension	Low levels of PA	High levels of PA	Average	*t*	*p*
Somatization	1.43 ± 0.54	1.35 ± 0.49	1.39 ± 0.52	8.31***	0.000
Obsessive-compulsive symptoms	1.82 ± 0.69	1.60 ± 0.61	1.71 ± 0.66	16.50***	0.000
Interpersonal sensitivity	1.66 ± 0.69	1.47 ± 0.58	1.57 ± 0.65	14.96***	0.000
Depression	1.61 ± 0.70	1.41 ± 0.56	1.52 ± 0.64	15.44***	0.000
Anxiety	1.56 ± 0.66	1.40 ± 0.54	1.48 ± 0.61	13.01***	0.000
Hostility	1.48 ± 0.61	1.36 ± 0.53	1.42 ± 0.58	10.06***	0.000
Phobia anxiety	1.45 ± 0.61	1.33 ± 0.52	1.40 ± 0.57	10.42***	0.000
paranoid ideation	1.50 ± 0.61	1.37 ± 0.52	1.44 ± 0.57	11.01***	0.000
psychoticism	1.48 ± 0.58	1.35 ± 0.49	1.42 ± 0.54	11.58***	0.000
Others	1.53 ± 0.61	1.40 ± 0.52	1.47 ± 0.57	11.76***	0.000

*** indicates 0.01 level of significance.

### Effects of different levels of PA on adolescents’ SA

An independent samples t-test were conducted to analyze the two variables, physical activity and school adaptation (Table 3). The results indicated that different levels of physical activity had statistically significant effects on both social competence and antisocial behavior. *Post hoc* comparisons revealed that high levels of physical activity were associated with higher scores in social competence compared low levels of physical activity, while high levels of physical activity were linked to lower scores antisocial behavior than low levels of physical activity.

**Table 3:** School adaptation (SA) scores of adolescents with different levels of PA (*x* ± *s*).

Dimension	Low levels of physical activity	High levels of physical activity	Overall average	*t*	*p*
Social capacity	5.82 ± 1.02	6.14 ± 1.05	5.97 ± 1.05	−15.27***	0.001
Antisocial behavior	1.24 ± 0.29	1.23 ± 0.34	1.24 ± 0.32	1.37***	0.000

*** indicates 0.01 level of significance.

### The mediating role of SA in PA and MH in adolescents

**Figure 1:**
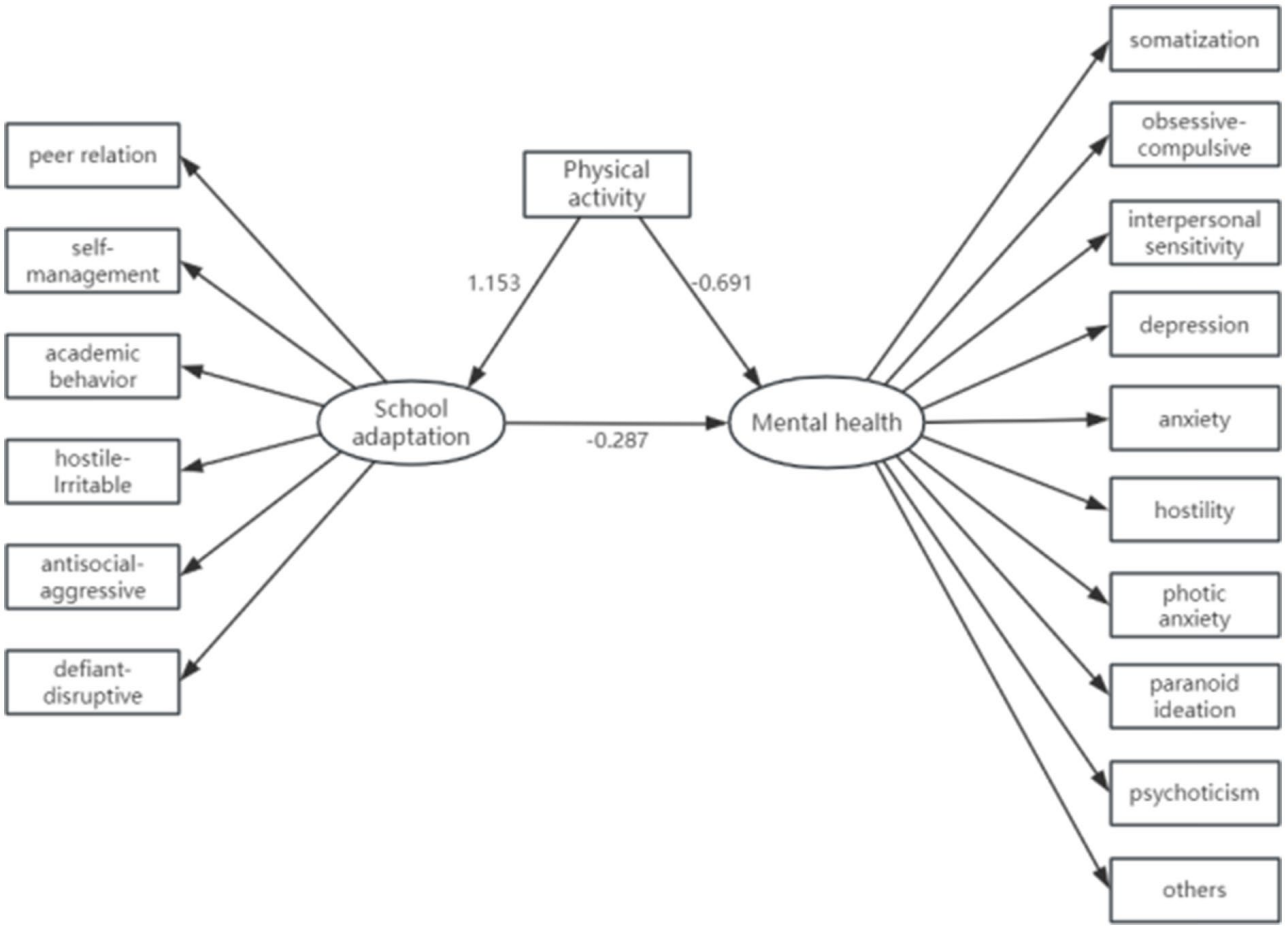
Mediating effect of SA on the relationship between PA and MH.

Mediation effects were assessed with school adaptation as the mediating variable, physical activity as the independent variable, and mental health status as the dependent variable. The structural Equation Model Fit Indicators were as follows: GFI = 0.950, RMSEA = 0.048, CFI = 0.972, NFI = 0.970, and SRMR = 0.077, indicating a well-fitting mode. The bootstrap method with 5,000 repeated samples was used for mediation effect test and 95% confidence interval estimation. The results showed (Table 4) that adolescent physical activity had a significant effect on mental health status (*β* = −0.691, *p* < 0.001). After including the mediator variable, school adaptation, the positive effect of adolescent physical activity on school adaptation was significant (*β* = 1.153, *p* < 0.001), as was the positive effect of school adaptation on mental health status (*β* = −0.287, *p* < 0.001). As shown in Table 5, the direct effect of physical activity on mental health was 0.011, while the indirect effect of physical activity on school adaptation versus school adaptation on mental health status was 0.024 (PA → SA → MH). Based on this, using structural equation modeling to construct mental health status, assuming school adaptation as the mediating variable, physical activity as the independent variable, and mental health status as the dependent variable. A mediated effects model was constructed, as illustrated in Figure 1.

**Table 4:** Mediating effects of SA on the relationship between PA and MH.

Route	β	S.E.	*p*
PA → SA	1.153	0.067	0.000
PA → MH	−0.691	0.070	0.000
SA → MH	−0.287	0.011	0.000

PA, physical activity; SA, school adaptation; MH, mental health status.

**Table 5:** Mediating effects of SA.

Route	S.E.	β	95%CI		
			Lower	Upper	*p*
PA → MH	0.011	−0.105	−0.127	−0.084	0.001
PA → SA → MH	0.024	−0.331	−0.381	−0.285	0.001

PA, physical activity; SA, school adaptation; MH, mental health status.

## Discussion

This study examined the relationships among physical activity (PA), school adaptation (SA) and mental health status (MH) in adolescents. It should be noted that the data collection period (2020–2021) coincided with significant disruptions to school routines and physical activity patterns due to pandemic restrictions, which may have influenced the observed relationships (Mengyao et al., 2024). The results of the study indicated that PA positively predicted adolescents’ MH, supporting hypothesis 1. Additionally, SA partially mediated the relationship between adolescents’ PA and MH, confirming hypothesis 2. Thus, PA is not only directly related to MH but also plays an indirect role through SA.

The findings showed that PA significantly affects MH, with adolescents with low levels of PA scoring higher across all nine dimensions of mental health status compared to those with high levels of PA. Research has shown that exercise increases the activity of endogenous opioids, such as beta-endorphins in the brain, which play a crucial role in regulating mood and emotional responses. This mechanism, known as the endorphin hypothesis, attributes the improvement in mood and reduction in anxiety following exercise to the release of beta-endorphins and their binding to brain receptors (Schoenfeld and Swanson, 2021). Furthermore, exercise enhances brain function and psychological well-being by increasing levels of brain-derived neurotrophic factors. Together, these mechanisms make exercise an effective means of alleviating mental health status (Xiao et al., 2025). Different types of exercise also have positive impact on MH, for instance, aerobic activities (Li et al., 2024; Meyer et al., 2024) such as running, swimming and cycling not only improves cardiovascular function but also enhances cognitive performance and emotional states; Yoga (Brodén et al., 2024) and Tai chi (Yang et al., 2024) can improve heart rate variability and reduces perceived stress levels, effectively alleviating symptoms of anxiety and depression. Participation in team sports (Karayel et al., 2024) significantly reduces the incidence of MH, improves social skills, and enhances the sense of social belonging and self-worth. Therefore, PA contributes to adolescents’ psychological health, allowing them to experience life more positively and maintain a stable mood.

PA also significantly affects adolescent SA. Adolescents with high levels of PA scored higher on social competence and lower on antisocial behavior than those with low levels of PA. Physical activity fosters one’s will power and helps students develop good habits and personality traits (Humińska-Lisowska, 2024). Participation in physical activities enables young people to establish positive relationships at school, promoting personality development, value formation, and improved adaptability (Cao et al., 2023). This is consistent with Wu et al. (2024) findings. Their study indicated that physical activity levels predict adolescents’ school adaptation, with a stronger association found at higher levels of physical activity. Wu et al. (2024) research also suggests that Research indicates that active participation in school sports activities can increase students’ positive attitudes toward school, enhance their sense of belonging to the school, and foster a sense of identification with the school’s culture. Engaging in organized sports fosters adaptation of adolescents to the school environment through social integration (Shamionov et al., 2021). Therefore, physical activities improve students’ adaptability, thus promoting their physical and psychological health.

The SA mediates the relationship between PA and MH in adolescents. This mediating effect is realized in a number of ways: physical activity enhances students’ ability to adapt to the school environment by increasing their self-regulation and peer integration (Li et al., 2020; Bai et al., 2022). The type of physical activity is also crucial, especially team sports, which provide structured opportunities for positive peer interaction (Clément et al., 2024; Chang et al., 2024). Through shared goals and cooperative tasks in physical activity, youth are able to make meaningful social connections and develop interpersonal skills that are transferable to the broader school environment (Long et al., 2021). These enhanced social competencies then further contribute to mental health by reducing isolation and increasing self-esteem. Aerobic activities like running, on the other hand, alleviate anxiety symptoms more directly through neurobiological mechanisms (Shannon et al., 2022). Exercises such as yoga and Tai Chi enhance emotional regulation and body awareness, which can be beneficial for students who face difficulties adapting to school due to anxiety (Valdesalici et al., 2024; Zou et al., 2018). In addition, peer relationships function as a parallel mediator (St Pierre et al., 2024), and individual adolescents who have negative peer relationships experience a negative impact on their psychological development and growth process (Lin et al., 2024). However, engaging in various types of PA can promote adolescents’ integration into the school environment, thereby reducing the incidence of MH. Therefore, establishing positive peer relationships can enhance students’ sense of school identity and improve their psychological health. The results of this study demonstrates that PA not only have a direct effect on adolescents’ MH, but also enhance psychological health by improving students’ school adaptability. Schools should maximize the mental health benefits of physical activity by implement customized physical activity programs that combine team activities with individual exercise to enhance social connections and meet specific psychological needs while promoting supportive peer networks.

### Strengths and limitations

The strengths of this study include first, the large sample size, obtained from 30 high schools in 10 districts of Shandong Province, which enhances the reliability and validity of the findings. Second, the results provide insights into the direct and indirect effects of PA on MH, offering practical references for the development of and interventions to improve the MH of adolescents. However, the study also has limitations. First, data collection for PA, SA, and MH relied on self-reports, which may be subject to self-report bias. Second, as a cross-sectional study, it could not establish causality. Given these limitations, future longitudinal studies with diverse sample sources and more comprehensive designs are needed to confirm these findings and explore their bidirectional relationships.

## Conclusion

In this cross-sectional sample, there was a direct effect of PA on adolescents’ MH, demonstrating that PA enhances their psychological health. Furthermore, SA mediates and moderates the relationship between PA and MH in adolescents. It is suggested that students’ physical quality and health level should be comprehensively improved by increasing physical education, enriching sports clubs, organizing extracurricular sports activities, monitor the sports effect by technical means, improve sports facilities and make regular evaluation feedback. So as to improve the school adaptability of teenagers, and finally achieve the all-round development of students physical and mental health.

## Data Availability

The original contributions presented in the study are included in the article/supplementary material, further inquiries can be directed to the corresponding author.
